# Twist tunable spin to charge conversion and valley contrasting effects in graphene on 2D transition metal dichalcogenides

**DOI:** 10.1038/s41598-025-23786-2

**Published:** 2025-11-07

**Authors:** I. Wojciechowska, A. Dyrdał

**Affiliations:** Faculty of Physics and Astronomy, Adam Mickiewicz University in Poznań, ul. Uniwersytetu Poznańskiego 2, 61-614 Poznań, Poland

**Keywords:** Materials science, Nanoscience and technology, Physics

## Abstract

We consider graphene deposited on monolayers of such transition-metal dichalcogenides like MoSe$\phantom{0}_2$, WSe$\phantom{0}_2$, MoS$\phantom{0}_2$, and WS$\phantom{0}_2$. Our key objective in this paper is to study the impact of relative twist angle between the monolayers on the proximity-induced spin-orbit interaction and orbital phenomena in graphene. To do this we use an effective model Hamiltonian for low-energy states, taken from the available literature. The linear response theory and Green function formalism are used to calculate analytical formulas for the spin Hall effect and nonequilibrium current-induced spin polarization in the systems. In addition, we also evaluate the valley Hall effect and nonequilibrium valley polarization, and focus especially on their dependence on the twist angle. We show that the valley Hall conductivity can achieve the quantum value equal to $\pm 2 e^2/h$.

## Introduction

Spin-orbit coupling (SOC) plays an essential role in the spin-dependent electronic properties of low-dimensional materials and spintronics applications. Although intrinsic SOC is weak in many individual two-dimensional (2D) crystals, it can be significantly enhanced through the so called proximity effects. Proximity-induced spin-orbit coupling arises when a 2D material with negligible intrinsic SOC, such as graphene, is placed in contact with a material possessing strong SOC, e.g., transition metal dichalcogenides (TMDCs) or topological insulators (TIs)^1–10^. The nature and magnitude of the induced SOC depend on the interlayer hybridization, symmetry, and stacking configuration.

The rapid advancement in fabrication of van der Waals (vdW) heterostructures^11–13^– combined with the discovery of correlated phases and superconductivity in magic-angle twisted bilayer graphene^5,14,15^– gave rise to the emergence of a specific area of spintronics, that is called twistronics^16^. Recent theoretical and experimental studies have already demonstrated that the twist angle between layers in vdW heterostructures strongly modifies interfacial interactions and allows for the control of band structure and symmetry-breaking effects^17–21^. In particular, rotational misalignment leads to the formation of moiré superlattices, which generate spatially varying interlayer hybridization and result in band reconstruction, mini-band formation, and altered Berry curvature distributions^5,22,23^. These moiré-induced effects directly influence the spin-orbit texture and can lift spin and valley degeneracies in a manner that is strongly dependent on the twist angle. As such, the twist angle serves as a critical tuning parameter for engineering proximity-induced SOC, potentially enabling phase transitions between distinct topological regimes^24–26^.

In this paper we consider the effect of relative twist between the monolayers of graphene and semiconducting TMDC in the H crystallographic phase on various transport characteristics of such heterostructures. To be more specific, we consider in detail such heterostructures like t-Gr/MoSe$\phantom{0}_2$, t-GrWSe$\phantom{0}_2$, t-Gr/MoS$\phantom{0}_2$, and t-Gr/WS$\phantom{0}_2$ (t-Gr abbreviates twisted graphene). The electronic band structures of these four heterostructures have been analysed recently within the density functional theory (DFT)^18,26,27^, where by numerical fitting procedure also the parameters describing effective low-energy Hamiltonians of graphene in the heterostructures have been determined. These effective Hamiltonians allow us to study analytically certain transport characteristics. Using the linear response theory and Green function formalism, we derived fully analytical formulas for the studied transport characteristics. characteristics. The paper is organized as follows. In Section "Model" we present the low energy effective Hamiltonian describing electronic properties of the twisted graphene deposited on 2H-TMDC monolayer, and twist-angle dependence of the parameters describing this Hamiltonian. We also present there the electronic band structure of graphene deposited on a semiconducting 2H-TMDC monolayer, and show how the twist angle changes the band structure and the corresponding spin-momentum locking. In Sections "Twist-angle tuneable spin-to-charge conversion" and "Valley Hall effect and valley Rashba-Edelstein effect controlled by the twist angle of graphene" we present results of our theoretical study of the spin-to-charge conversion and valley contrasting effects obtained within the Green function formalism and linear response theory. In Sec. "Twist-angle tuneable spin-to-charge conversion" it is shown how the twist angle modifies the spin Hall effect and the spin-to-charge conversion phenomena (i.e., the Rashba-Edelstein effect), while in Sec. "Valley Hall effect and valley Rashba-Edelstein effect controlled by the twist angle of graphene" the valley Hall effect and valley nonequilibrium spin polarization are presented and discussed. Finally, the summary and discussion of our results are presented in Sec. "Discussion and summary".

**Fig. 1:**
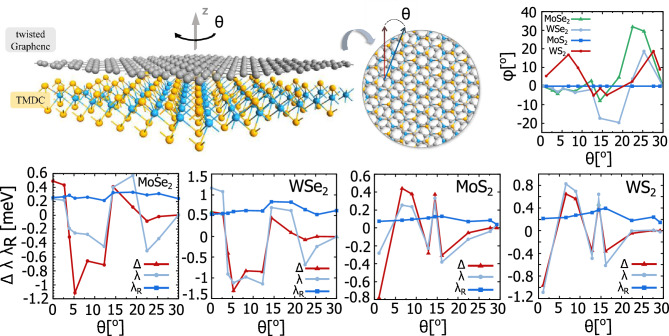
Schematic picture of graphene twisted by the angle $\theta$ with respect to the monolayer of TMDC (side and top views) and parameters defining Hamiltonian (1) as a function of the twist-angle $\theta$ for the four semiconducting transition metal dichalcogenides: MoSe$\phantom{0}_2$, WSe$\phantom{0}_2$, MoS$\phantom{0}_2$, and WS$\phantom{0}_2$. Data are taken from Ref.(^18^).

## Model

We consider graphene deposited on a monolayer of one of the semiconducting transition metal dichalcogenices, like MoSe$\phantom{0}_{2}$, WSe$\phantom{0}_{2}$, MoS$\phantom{0}_{2}$ and WSe$\phantom{0}_{2}$. The effective Hamiltonian describing low-energy electronics states around the K and K’ points of the Brillouine zone of graphene in proximity to semiconducting TMDCs in the 2H phase takes the following form^18,28–31^:

1 $$\begin{aligned} \hat{H}^{\nu } = {\hat{H}}_{\scriptscriptstyle {0}}^{\nu }+{\hat{H}}_{\scriptscriptstyle {\Delta }}+{\hat{H}}_{\scriptscriptstyle {I}}^{\nu }+{\hat{H}}_{\scriptscriptstyle {R}}^{\nu }\, , \end{aligned}$$

where the individual terms of the above Hamiltonian read:

2 $$\begin{aligned} \hat{H}_{\scriptscriptstyle {0}}^{\nu } =&\upsilon (\nu k_x \hat{\sigma }_x - k_y \hat{\sigma }_y)\otimes \hat{s}_0, \end{aligned}$$

3 $$\begin{aligned} \hat{H}_{\scriptscriptstyle {\Delta }} =&\Delta \hat{\sigma }_z \otimes \hat{s}_0, \end{aligned}$$

4 $$\begin{aligned} \hat{H}_{\scriptscriptstyle {I}}^{\nu } =&\nu (\lambda _{\text {I}}^\text {A} \hat{\sigma }_{+}+\lambda _{\text {I}}^\text {B} \hat{\sigma }_{-})\otimes \hat{s}_z, \end{aligned}$$

5 $$\begin{aligned} \hat{H}_{\scriptscriptstyle {R}}^{\nu } =&-\lambda _{\text {R}}\text {e}^{-\text {i}\varphi \frac{\hat{s}_z}{2}}(\nu \hat{\sigma }_x \otimes \hat{s}_y + \hat{\sigma }_y \otimes \hat{s}_x)\text {e}^{\text {i}\varphi \frac{\hat{s}_z}{2}} \end{aligned}$$

We have used above the following notation: $\nu$ as index stands for K and K’, while $\nu$ as a factor is defined as $\nu = 1(-1)$ for the K(K’) valleys, respectively, $k_{x,y}$ are the components of the wavevector, i.e., $\textbf{k} = (k_x, k_y)$ and $k_{x}^{2} + k_{y}^{2} = k^2$; the matrices $\hat{\sigma }_{0}, \hat{{\varvec{\sigma }}} = (\hat{\sigma }_{x}, \hat{\sigma }_{y}, \hat{\sigma }_{z})$ denote the identity matrix and Pauli matrices acting in the pseudospin space, whereas $\hat{s}_{0}, \hat{\textbf{s}} = (\hat{s}_{x}, \hat{s}_{y}, \hat{s}_{z})$ define identity and Pauli matrices acting in the spin space. The matrices $\hat{\sigma }_{\pm }$ are defined as $\hat{\sigma }_{\pm } = (\hat{\sigma }_{z} \pm \hat{\sigma }_{0})/2$. The first term of the Hamiltonian (1), captures the orbital physics of pristine graphene ($\upsilon = \hbar v_{\textrm{F}}$ with $v_{\textrm{F}}$ denoting Fermi velocity). The second term of  (1), $\hat{H}_{\Delta }$, describes the staggered potential arising due to sublattice symmetry breaking. The last two terms in Eq. (1) describe two possible components of the spin-orbit coupling, that may appear in the structure: the so-called intrinsic spin-orbit interaction and Rashba coupling. The intrinsic SOC, given by Eq. (4), has a sublattice dependent form with the copuling parameters $\lambda _{I}^{A,B}$. The DFT data used in our considerations^18^ indicate that the intrinsic SOC is of the valley-Zeeman type, accordingly $\lambda _{I}^{A} = - \lambda _{I}^{B} = \lambda$. The Rashba spin-orbit interaction is given by the generalised form that contains two parameters: the coupling amplitude, $\lambda _{R}$, and the Rashba angle, $\phi$, determining the spin-momentum locking in the system.

**Fig. 2:**
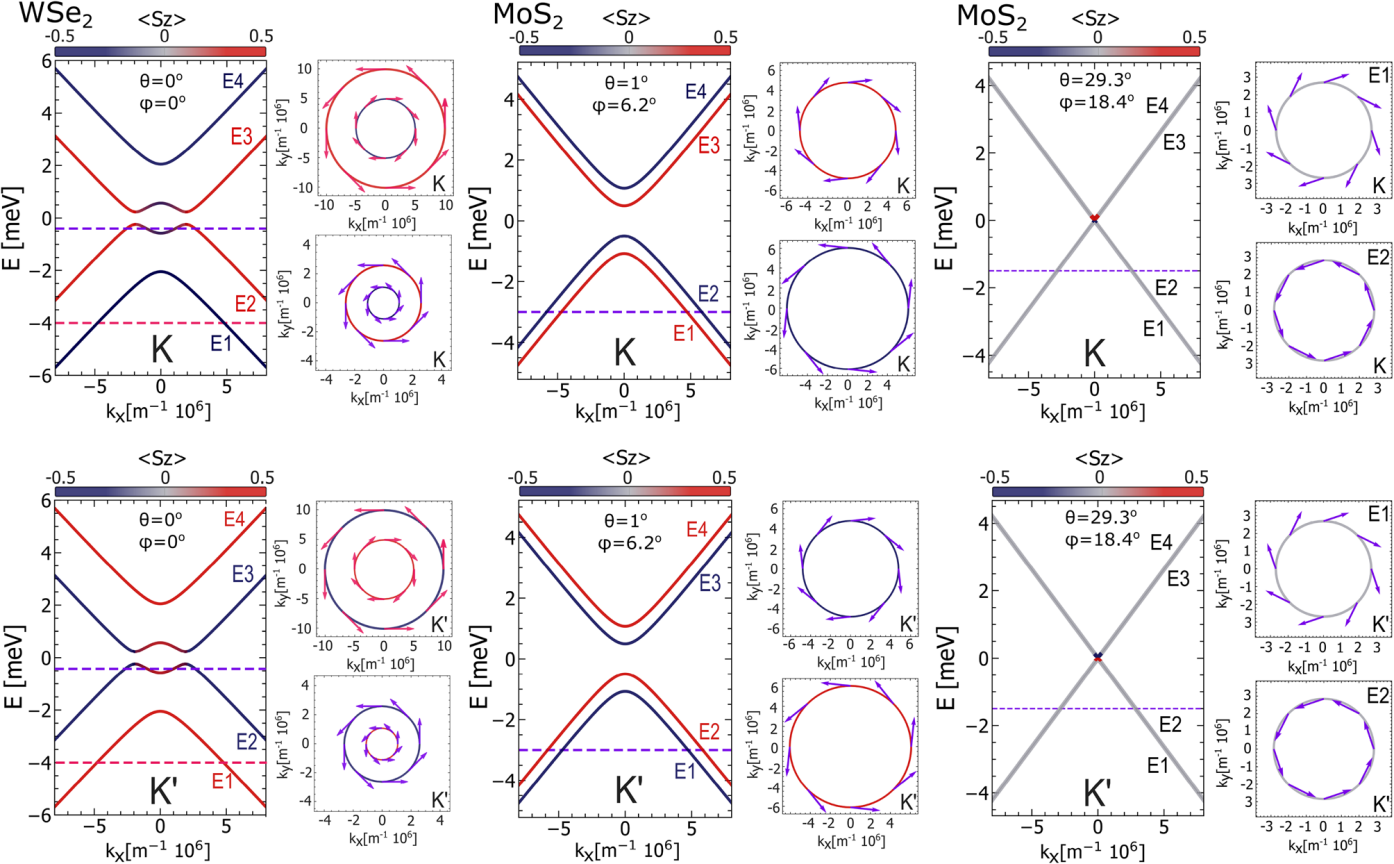
The energy band structure of t-Gr/WSe$\phantom{0}_2$ plotted for the twist angle $\theta = 0 ^{\circ }$ and the Rashba angle $\phi = 0 ^{\circ }$, and of t-Gr/MoS$\phantom{0}_2$ plotted for $\theta = 1^{\circ }$ and the Rashba angle $\phi = 6.2 ^{\circ }$, as well as for $\theta = 29.3^{\circ }$ and the Rashba angle $\phi = 18.4 ^{\circ }$. The colour of the band lines corresponds to the $s_z$ spin expectation value, whereas the in-plain spin expectation values have been indicated on energy contours. The values of parameters: $\lambda$, $\lambda _{R}$, $\phi _R$ and $\Delta$ for the certain twist angle, $\theta$, are taken from Fig 1, the $\upsilon$ parameter is equal $5.414\cdot 10^{-10}$ eVm for WSe$\phantom{0}_2$ and $4.356\cdot 10^{-10}$ eVm for MoS$\phantom{0}_2$.

The eigenvalues of Hamiltonian (1) take the following form:

6 $$\begin{aligned} E_{1,4}&= \mp \sqrt{k^2\upsilon ^2 + \Delta ^2 + \lambda ^2 + 2\lambda _{\scriptscriptstyle {R}}^2 + 2\xi }, \end{aligned}$$

7 $$\begin{aligned} E_{2,3}&= \mp \sqrt{k^2\upsilon ^2 + \Delta ^2 + \lambda ^2 + 2\lambda _{\scriptscriptstyle {R}}^2 - 2\xi }, \end{aligned}$$

where $\xi = \sqrt{(\Delta \lambda + \lambda _{\scriptscriptstyle {R}}^2)^2 + k^2\upsilon ^2(\lambda ^2 + \lambda _{\scriptscriptstyle {R}}^2)^2}$. Importantly, all the parameters defining the eigenvalues $E_{1-4}$ depend on material parameters and are also strongly dependent on the twist angle $\theta$, as presented in Fig. 1. In Fig. 2 the low-energy electronic states of graphene deposited on a single-layer of TMDCs are presented for fixed values of the twist angle. The expectation values of the spin at certain wavevectors are also presented there. The selected examples reflect the possible behaviour of the eigenstates, that one can obtain for a fixed twist angle. Accordingly, the low-energy bands of t-Gr/WSe$\phantom{0}_2$ for the twist angle $\theta = 0^{\circ }$ and Rashba angle $\phi = 0 ^{\circ }$ are strongly spin-splitted with a characteristic ’Mexican hat’ shape of the top-most valence and bottom-most conduction bands, which is typical for the case when the staggered potential, $\Delta$, dominates over the intrinsic spin-orbit coupling strength, $\lambda$. In turn, for t-Gr/MoS$\phantom{0}_2$ structure with the $\theta$ equal 1$\phantom{0}^{\circ }$ and $29.3^{\circ }$ one finds $|\lambda | < |\Delta |$ and the spin splitting occurs vertically (the energy bands have only single extremal point at $k = 0$). Note that the vanishing of staggered potential and intrinsic SOC leads to the almost degenerate bands and vanishing of the bandgap (this is the case of t-Gr/MoS$\phantom{0}_2$ for the twist angle $\theta = 29.3^{\circ }$ ).

## Twist-angle tuneable spin-to-charge conversion

The intentional twist of graphene with respect to TMDC allows a significant tuning of spin proximity effects and, consequently, enables tuning of the spin-to-charge conversion effects. In this section we analyse behaviour of the two most important effects for spin-orbitronics. i.e., spin Hall effect and current-induced spin polarization, also known as Rashba-Edelstein effect (REE) or inverse spin galvanic effect^32–39^. Generally, one can write the total spin Hall conductivity (SHC) in the system under consideration as a sum of the spin Hall conductivities associated with the two inequivalent valleys, *K* and $K'$.

8 $$\begin{aligned} \sigma _{SH} = \sigma _{xy}^{s_z\,K} + \sigma _{xy}^{s_z\,K'}. \end{aligned}$$

Using the linear response theory and Green function formalism, the spin Hall conductivity in the dc limit for the valley $\nu$ ($\nu = K,K'$) can be calculated from the formula^40–43^:

9 $$\begin{aligned} \sigma _{yx}^{s_{z}\,\nu } = \lim _{\omega \rightarrow 0} \frac{e^{2} \hbar }{\omega } \int \frac{d \varepsilon }{2\pi } \int \frac{d^{2}\textbf{k}}{(2\pi )^{2}} \textrm{Tr} \left[ \hat{j}_{y}^{s_z\,\nu } G^{\nu }_{\textbf{k}}(\varepsilon + \omega ) \hat{\upsilon }_{x}^{\nu }G^{\nu }_{\textbf{k}}(\varepsilon ) \right] \end{aligned}$$

where the spin current density operator is defined as $\hat{j}_{y}^{s_z\,\nu } = \frac{1}{2}[\hat{\upsilon }_{y}^{\nu },\hat{s}_{z}]_{+}$, with the velocity, $\hat{\upsilon }_{\alpha }^{\nu }$, and spin, $\hat{s}_{z}$, operators defined as $\hat{\upsilon }_{\alpha }^{\nu } = \frac{1}{\hbar } \frac{\partial \hat{H}^{\nu }}{\partial k_{\alpha }}$ ($\alpha = {x,y}$), $\hat{s}_{z} = \frac{\hbar }{2}\hat{\sigma _0} \otimes \hat{s}_z$, respectively. $G_{\textbf{k}}^{\nu }(\varepsilon )$ is the casual Green’s function defined as $G_{\textbf{k}}^{\nu }(\varepsilon ) = [(\varepsilon + \mu + i\delta \textrm{sign}(\varepsilon ))\sigma _{0} \otimes \hat{s}_{0} - \hat{H}^{\nu }]^{-1}$, where $\mu$ denotes the chemical potential and $\delta \rightarrow 0^{+}$ (as we consider the clean limit).

Taking into account the two possible situations corresponding to the two possible shapes of the spin-splitted bands (i.e., ’Mexican hat’ or ’vertical like’), we find analytical expressions for the spin Hall conductivity in all the energy regions. In general, in the system under consideration the formulas for the spin Hall conductivity can be defined in seven distinct energy regions. Accordingly, when the bands are spin-splitted in a vertical-like way, that is when $0<|\lambda | < |\Delta |$, one finds the following expressions valid for the specific ranges of the Fermi energy. Thus, when the Fermi energy crosses both conduction or both valence bands, i.e., when $\left| \mu \right| > \sqrt{(\Delta +\lambda )^2+4\lambda _{\scriptscriptstyle {R}}^2}$ the formula for spin Hall conductivity takes the form

10 $$\begin{aligned} \sigma _{SH} = \mp \frac{\Delta \lambda ^3 }{2 \left( \lambda ^2+\lambda _{\scriptscriptstyle {R}}^2\right) } \left( \frac{1}{\zeta _{\scriptscriptstyle {+}}}-\frac{1}{\zeta _{\scriptscriptstyle {-}}}\right) \pm \frac{\lambda _{\scriptscriptstyle {R}}^2}{4 \left( \lambda ^2+\lambda _{\scriptscriptstyle {R}}^2\right) ^2}\left[ \lambda ^2\left( \frac{\chi _{\scriptscriptstyle {1+}}}{\zeta _{\scriptscriptstyle {+}}} - \frac{\chi _{\scriptscriptstyle {1-}}}{\zeta _{\scriptscriptstyle {-}}} \right) + \lambda _{\scriptscriptstyle {R}}^2\left( \frac{\chi _{\scriptscriptstyle {2+}}}{\zeta _{\scriptscriptstyle {+}}} - \frac{\chi _{\scriptscriptstyle {2-}}}{\zeta _{\scriptscriptstyle {-}}} \right) \right] , \end{aligned}$$

where we used the following notation:

11 $$\begin{aligned}&\chi _{\scriptscriptstyle {1\pm }}=\pm 2\eta + \mu ^{2}-2\lambda ^{2}+(\lambda -\Delta )^{2}, \end{aligned}$$

12 $$\begin{aligned}&\chi _{\scriptscriptstyle {2\pm }}=\pm 2\eta + \mu ^{2}-2\lambda ^{2}-(\lambda -\Delta )^{2}, \end{aligned}$$

13 $$\begin{aligned}&\zeta _{\scriptscriptstyle {\pm }}=\sqrt{\kappa \pm 2\eta (\lambda ^{2}+\lambda _{R}^{2})}, \end{aligned}$$

14 $$\begin{aligned}&\eta =\sqrt{\mu ^{2}(\lambda ^{2}+\lambda _{R}^{2})-(\lambda -\Delta )^{2}\lambda _{R}^{2}}, \end{aligned}$$

15 $$\begin{aligned}&\kappa =(\Delta \lambda + \lambda _{R}^{2})^{2} + (\mu ^{2}+\lambda ^{2}-\Delta ^{2})(\lambda ^{2}+\lambda _{R}^{2}). \end{aligned}$$

When Fermi energy crosses only one valence or one conduction band, $\left| \lambda -\Delta \right| < \left| \mu \right| \le \sqrt{(\Delta +\lambda )^2+4\lambda _{\scriptscriptstyle {R}}^2}$, one finds

16 $$\begin{aligned} \sigma _{SH} = \pm \frac{\Delta \lambda ^3 }{2 \zeta _{\scriptscriptstyle {+}} \left( \lambda ^2+\lambda _{\scriptscriptstyle {R}}^2\right) } \mp \frac{\lambda _{\scriptscriptstyle {R}}^2}{4 \zeta _{\scriptscriptstyle {+}} \left( \lambda ^2+\lambda _{\scriptscriptstyle {R}}^2\right) ^2}\left( \lambda ^2 \chi _{\scriptscriptstyle {1+}} + \lambda _{\scriptscriptstyle {R}}^2 \chi _{\scriptscriptstyle {2-}}\right) \hspace{4cm} \nonumber \\ \hspace{2cm}\pm \frac{\lambda }{2\left( \lambda ^2+\lambda _{\scriptscriptstyle {R}}^2\right) ^2 \left| \Delta \lambda +\lambda _{\scriptscriptstyle {R}}^2\right| }\bigg [\lambda \lambda _{\scriptscriptstyle {R}}^2\left( \Delta ^2 - \lambda \Delta - \lambda ^2 \right) - \Delta \lambda ^4 + \lambda _{\scriptscriptstyle {R}}^4\left( \Delta - 2 \lambda \right) \bigg ]. \end{aligned}$$

For Fermi energy located between the top-most valence or bottom-most conduction band minima and maxima, i.e., ${-\left| \lambda -\Delta \right| \sqrt{\frac{\lambda _{R}^{2}}{\lambda _{R}^{2}+\lambda ^{2}}} \le \mu \le \left| \lambda -\Delta \right| \sqrt{\frac{\lambda _{R}^{2}}{\lambda _{R}^{2}+\lambda ^{2}}} }$ (this is when the bands have Mexican hat shape) one gets

17 $$\begin{aligned} \sigma _{SH} = \pm \frac{\Delta \lambda ^3 }{2 \left( \lambda ^2+\lambda _{\scriptscriptstyle {R}}^2\right) } \left( \frac{1}{\zeta _{\scriptscriptstyle {+}}}-\frac{1}{\zeta _{\scriptscriptstyle {-}}}\right) \mp \frac{\lambda _{\scriptscriptstyle {R}}^2}{4 \left( \lambda ^2+\lambda _{\scriptscriptstyle {R}}^2\right) ^2}\left[ \lambda ^2\left( \frac{\chi _{\scriptscriptstyle {1+}}}{\zeta _{\scriptscriptstyle {+}}} - \frac{\chi _{\scriptscriptstyle {1-}}}{\zeta _{\scriptscriptstyle {-}}} \right) + \lambda _{\scriptscriptstyle {R}}^2\left( \frac{\chi _{\scriptscriptstyle {2+}}}{\zeta _{\scriptscriptstyle {+}}} - \frac{\chi _{\scriptscriptstyle {2-}}}{\zeta _{\scriptscriptstyle {-}}} \right) \right] . \end{aligned}$$

Finally, for $-\left| \lambda -\Delta \right| \sqrt{\frac{\lambda _{R}^{2}}{\lambda _{R}^{2}+\lambda ^{2}}}<\mu <\left| \lambda -\Delta \right| \sqrt{\frac{\lambda _{R}^{2}}{\lambda _{R}^{2}+\lambda ^{2}}}$ (that is for the Fermi energy located inside the energy gap) the spin Hall conductivity vanishes, $\sigma _{SH} = 0$. Here, it should be also stressed that, according to the data obtained based on DFT calculations, the above formulas have been obtained assuming that $\Delta \lambda > 0$. Moreover, when the top-most valence and bottom-most conduction bands change their shapes from the Mexican-hat-like dispersion to the dispersion with only a single extremal point, the energy range ${-\left| \lambda -\Delta \right| \sqrt{\frac{\lambda _{R}^{2}}{\lambda _{R}^{2}+\lambda ^{2}}} \le \varepsilon \le \left| \lambda -\Delta \right| \sqrt{\frac{\lambda _{R}^{2}}{\lambda _{R}^{2}+\lambda ^{2}}} }$ vanishes and the energy gap is defined in the energy range $-|\lambda - \Delta |< \mu < |\lambda - \Delta |$. This happens when $\lambda \ll \lambda _{R}$.

**Fig. 3:**
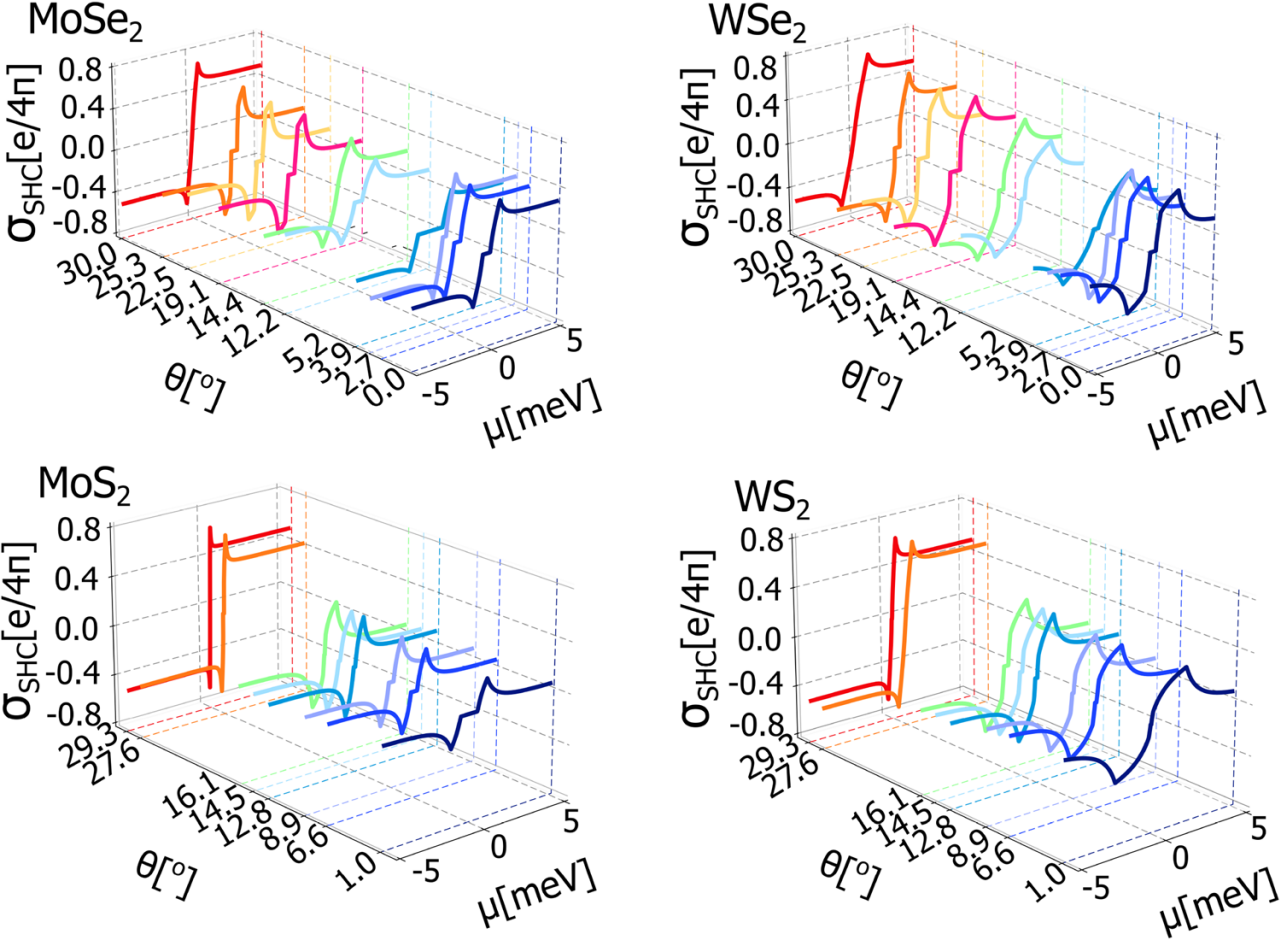
The spin Hall conductivity of graphene deposited on semiconducting TMDC monolayer (i.e., t-Gr/MoSe$\phantom{0}_{2}$, t-Gr/WSe$\phantom{0}_{2}$, t-Gr/MoS$\phantom{0}_{2}$ and t-Gr/WSe$\phantom{0}_{2}$) as a function of the Fermi energy, $\mu$, for certain twist angle, $\theta$, between graphene and TMDC. The values of parameters $\lambda$, $\lambda _{R}$, $\phi _R$ and $\Delta$ for the certain twist angle, $\theta$, are taken from Fig 1, the $\upsilon$ parameter is equal $5.414\cdot 10^{-10}$ eVm for WSe$\phantom{0}_2$, $4.348\cdot 10^{-10}$ eVm for WS$\phantom{0}_2$, $5.413\cdot 10^{-10}$ eVm for MoSe$\phantom{0}_2$, and $4.356\cdot 10^{-10}$ eVm for MoS$\phantom{0}_2$.

Figure 3 shows the spin Hall conductivity plotted as a function of the Fermi energy, $\mu$, for specific values of the twist angle,$\theta$, in the range between $0^{\circ }$ and $30^{\circ }$, and for t-Gr/MoSe$\phantom{0}_{2}$, t-Gr/WSe$\phantom{0}_{2}$, t-Gr/MoS$\phantom{0}_{2}$ and t-Gr/WSe$\phantom{0}_{2}$. One can easily note that the twist of graphene layer with respect to the TMDC monolayer can significantly modulate the spin Hall conductivity. We also note that the spin Hall conductivity behaves antisymmetric with respect to the sign of Fermi energy, and reveals a sharp peak when Fermi energy crosses the minimum of the second conduction band and maximum of the bottom-most valence band. Such a behaviour is expected in proximitized graphene systems with Rashba SOC. However, the spin Hall conductivity does not depend on the Rashba angle $\phi$ (what clearly follows from the obtained analytical formulas). It should also be stressed that in the considered heterostructures, the valley-Zeeman and Rashba SOC are responsible for the spin Hall effect. However, as the spin $s_z$ is not conserved, the spin Hall conductivity is not observed when Fermi energy is in the energy gap, i.e., there is no quantum spin Hall insulator state^44–48^ as the contribution from the Fermi see to the spin Hall conductivity vanishes.

Based on the plots presented in Fig. 1, it is seen that the fitted Rashba SOC amplitude, $\lambda _{R}$, is rather not affected by the twist angle. In contrast, the staggered potential, $\Delta$, and valley-Zeeman SOC parameter, $\lambda$, can significantly change with the twist. Accordingly, the change of $\Delta$ and $\lambda$ with the twist angle controls the width of the energy gap and consequently sets a specific energy range where we do not observe any spin current. From the plots presented in Fig. 3 one can note that SHC achieves the largest values for the twist angles, $\theta$, equal $30^{\circ }$ (MoSe$\phantom{0}_{2}$,WSe$\phantom{0}_{2}$) and $29.3^{\circ }$ (MoSe$\phantom{0}_{2}$,WSe$\phantom{0}_{2}$). These angles correspond to the case when the parameter $\lambda$ tends to zero, which is caused by emerging mirror plane symmetry. Under these circumstances the Rashba SOC dominates in the system.

**Fig. 4:**
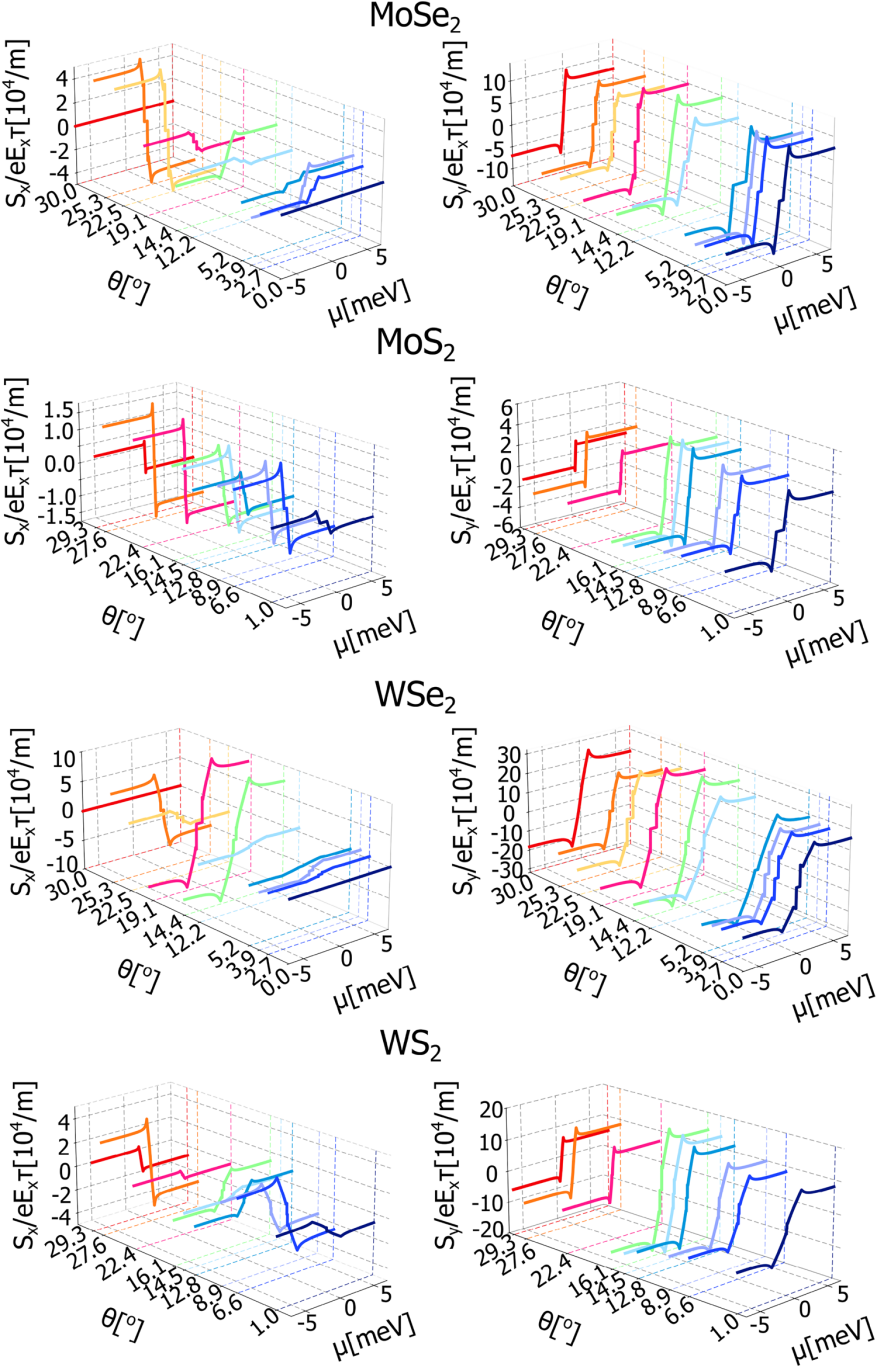
The *x* and *y* components of nonequilibrium spin polarization plotted as a function of Fermi energy, $\mu$, for specific values of the twist angle, $\theta$. The values of parameters $\lambda$, $\lambda _{R}$, $\phi _R$ and $\Delta$ for the certain twist angle, $\theta$, are taken from Fig 1, the $\upsilon$ parameter is equal $5.414\cdot 10^{-10}$ eVm for WSe$\phantom{0}_2$, $4.348\cdot 10^{-10}$ eVm for WS$\phantom{0}_2$, $5.413\cdot 10^{-10}$ eVm for MoSe$\phantom{0}_2$, and $4.356\cdot 10^{-10}$ eVm for MoS$\phantom{0}_2$.

The nonequilibrium spin polarization arising in the system as a consequence of the Rashba-Edelstein effect (REE) can be calculated within the Green function formalism, too. Accordingly, the components of the nonequilibrium spin densities can be calculated starting from the expression:

18 $$\begin{aligned} S_{\alpha } = S_{\alpha }^{K}+S_{\alpha }^{K'} \end{aligned}$$

where^40,41,49,50^

19 $$\begin{aligned} S_{\alpha }^{\nu } = \lim _{\omega \rightarrow 0} \frac{e E_{x} \hbar }{\omega } \int \frac{d^{2}\textbf{k}}{(2\pi )^{2}} \int \frac{d \varepsilon }{2\pi } \textrm{Tr} \left[ \hat{s}_{\alpha } G^{\nu }_{\textbf{k}}(\varepsilon + \omega ) \hat{\upsilon }_{x}^{\nu }G^{\nu }_{\textbf{k}}(\varepsilon ) \right] . \end{aligned}$$

The above expression in the dc-limit takes the form:

20 $$\begin{aligned} S_{\alpha }^{\nu } = \dfrac{e E_{x}\hbar }{2\pi }\int \,\frac{d^{2}\textbf{k}}{(2\pi )^{2}}\textrm{Tr} \left[ \hat{s}_{\alpha }G_{\textbf{k}}^{R\,\nu }\hat{\upsilon }_{x}^{\nu }G_{\textbf{k}}^{A\,\nu }\right] , \end{aligned}$$

that contains contribution from the states at the Fermi level. Also in this case we obtained fully analytical formulas, however they are rather cumbersome, so we decided to show only the numerical results, as presented in Fig. 4. As one can note, the nonequilibrium spin polarization for each of the considered system contains two nonzero components, that are proportional to the external electric field, $E_x$ and relaxation time, $\tau$. Both nonzero components are oriented in the plain of the structure, i.e., the *y*-component is oriented perpendicularly to the external electric field and the *x*-component is oriented parallel to the electric field. The component parallel to the external electric field, recently called the unconventional Rashba Edelstein effect^18,51^, is quite naturally expected in systems with more complicated forms of spin-orbit coupling^52,53^. In the heterostructures considered in this paper, the nonzero $S_{x}$ component of spin polarization is a simple consequence of the nonzero Rashba angle, $\phi$, that governs the spin-momentum locking and deflects the expectation value of spin out of the perpendicular orientation to the quasiparticle momentum ($\phi = 0^{\circ }$). As the maximum Rashba angle $|\phi |$, appearing for a certain twist angle, is about $20^{\circ }$ for t-GR/WS$\phantom{0}_2$ and t-Gr/WSe$\phantom{0}_2$ and $30^{\circ }$ for MoSe$\phantom{0}_2$, thus the *x* component of the spin polarization is always smaller than the *y* component. Fig. 4 also clearly shows that both $S_{x}$ and $S_{y}$ components behave anti-symmetrically with respect of the sign reversal of the Fermi energy, and their magnitudes are strongly modulated by the twist angle. Importantly, the sign of the $S_{x}$ component can be changed for those twist angles for which the Rashba angle is negative.

## Valley Hall effect and valley Rashba-Edelstein effect controlled by the twist angle of graphene

The graphene based heterostructures with the two inequivalent valleys at K and K’ points of the Graphene’s Brillouine zone provide an excellent platform for exploring the valley physics and valley contrasting phenomena. The corresponding research area, i.e., valleytronics, is focused on an active use of the additional electron degree of freedom due to valleys (local minimum/extremum in the electronic band structure). The possibility of usage in data storage and logic devices another degree of freedom, in addition to the charge and spin ones, is very promising and has focused a lot of attention in recent years by theory and experimental groups.

**Fig. 5:**
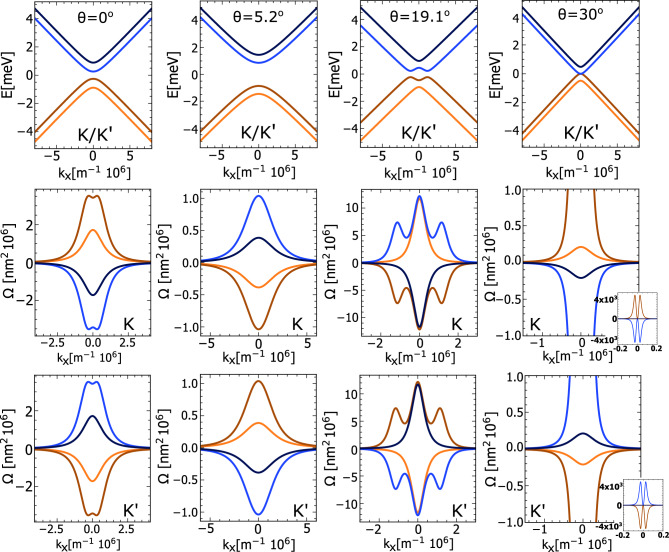
The band structure of t-Gr/MoS$\phantom{0}_2$ for the four selected twist angles, $\theta$, and the associated Berry curvatures plotted around the K and K’ points, respectively. The colours of individual Berry curvatures correspond to the associated energy band. The values of parameters $\lambda$, $\lambda _{R}$, $\phi _R$ and $\Delta$ for the certain twist angle, $\theta$, are taken from Fig 1, the $\upsilon$ parameter is equal $4.356\cdot 10^{-10}$ eVm.

**Fig. 6:**
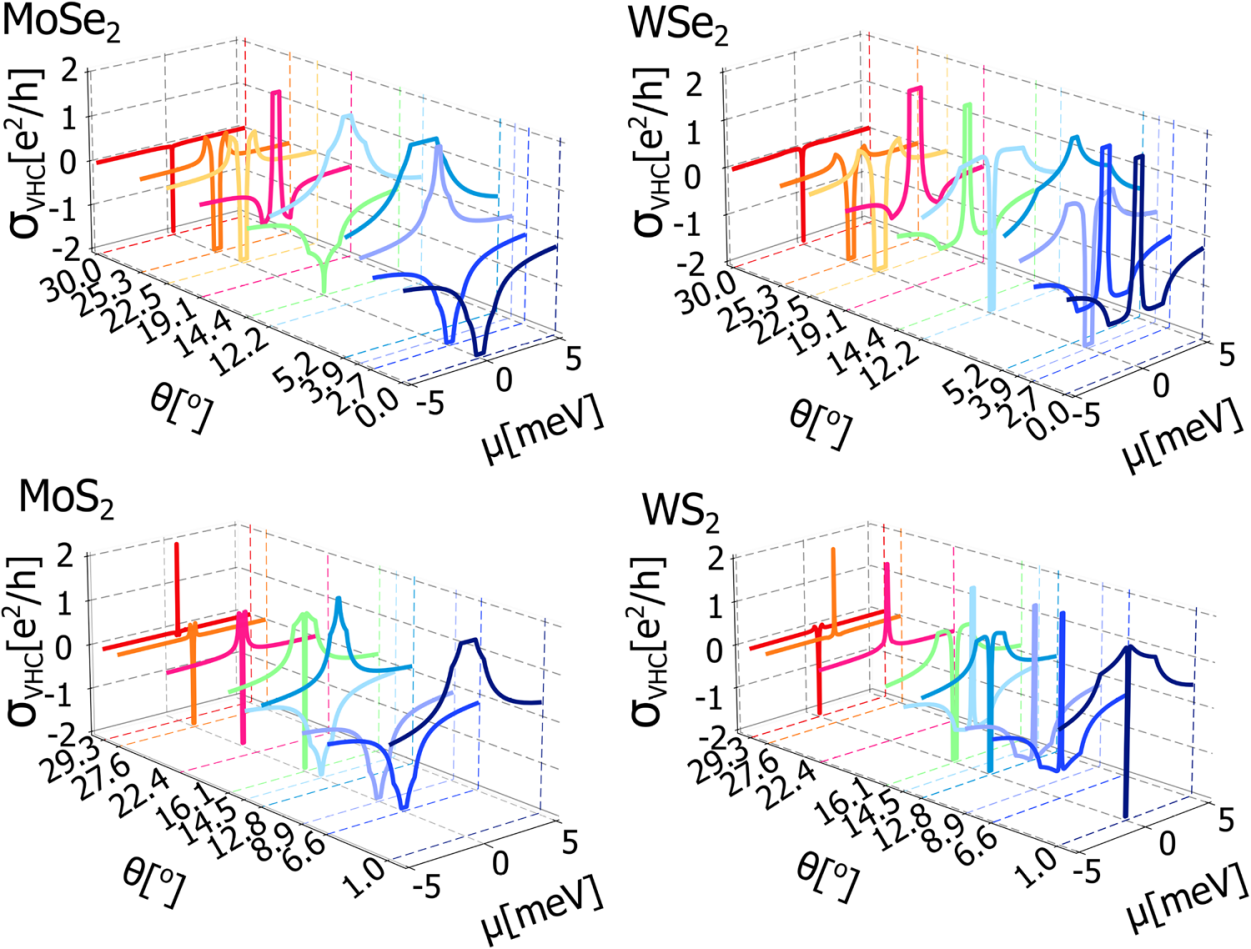
Valley Hall conductivity of graphene deposited on considered semiconducting TMDC monolayer as a function of the Fermi energy, $\mu$, for certain twist angle between graphene and TMDC, $\theta$. The values of parameters $\lambda$, $\lambda _{R}$, $\phi _R$ and $\Delta$ for the certain twist angle, $\theta$, are taken from Fig 1, the $\upsilon$ parameter is equal $5.414\cdot 10^{-10}$ eVm for WSe$\phantom{0}_2$, $4.348\cdot 10^{-10}$ eVm for WS$\phantom{0}_2$, $5.413\cdot 10^{-10}$ eVm for MoSe$\phantom{0}_2$, and $4.356\cdot 10^{-10}$ eVm for MoS$\phantom{0}_2$.

Importantly, the relative twist of graphene with respect to the substrate can substantially change also the orbital and valley dependent phenomena. Here we focus on two such phenomena, i.e., on the valley Hall effect and valley Rashba-Edelstein effect. The valley Hall effect can be defined as^42,43,54^:

21 $$\begin{aligned} \sigma _{VH} = \sigma _{xy}^{s_z\,K} - \sigma _{xy}^{s_z\,K'} \end{aligned}$$

where the charge conductivity is given by the general expression:^40,41^

22 $$\begin{aligned} \sigma _{yx}^{\nu } = \lim _{\omega \rightarrow 0} \frac{e^{2} \hbar }{\omega } \int \frac{d \varepsilon }{2\pi } \int \frac{d^{2}\textbf{k}}{(2\pi )^{2}} \textrm{Tr} \left[ \hat{\upsilon }_{y}^{\nu } G^{\nu }_{\textbf{k}}(\varepsilon + \omega ) \hat{\upsilon }_{x}^{\nu }G^{\nu }_{\textbf{k}}(\varepsilon ) \right] . \end{aligned}$$

Here, one should mention, that the valley Hall conductivity defined above is a special case of the valley orbital Hall effect^55–57^. The theory of orbital Hall effect provides not only a more general approach, but also offers a more accurate description of transport, being able to capture orbital flux in centrosymmetric systems, intervalley-coupled systems, and also serving as a precursor of spin Hall effects and quantum Hall effects^58–61^. The consistent theory of the orbital Hall effects is still under development, thus for the purpose of this paper we keep the above definition of valley Hall effect, whereas the more detailed discussion of the orbital effects in the graphene based twisted structures will be presented elsewhere.

In the case of intrinsic valley Hall effect, Eq. (22) leads to the expression that relates the conductivity at the $\nu$th valley to the corresponding Berry curvatures $\Omega _{j}$:

23 $$\begin{aligned} \sigma _{xy}^{\nu } = \frac{e^{2}}{\hbar } \sum _j \int \frac{d^{2} \textbf{k}}{(2\pi )^{2}} \Omega _{j}^{\nu } f(E_{j}) \end{aligned}$$

where $f(E_{j})$ is the Fermi-Dirac distribution function for the *j*-th subband at the $\nu$-th valley, and $\Omega _{j}^{\nu }$ is the corresponding Berry curvature. The total Chern number for the specific band is: $\Omega _{j} = \Omega _{j}^{K} + \Omega _{j}^{K'}$. Accordingly, one can clearly see that the valley Hall effect can appear even in the case of vanishing Berry curvature, provided it is nonzero locally at distinct valleys. This situation occurs in the heterostructures under consideration. In Fig. 5 the Berry curvature integrated over the azimutal angle for graphene deposited on MoS$\phantom{0}_2$ is plotted in the vicinity of the K and K’ points and for certain twist angles in the range between $0^{\circ }$ and $30^{\circ }$. The twist angle substantially modulate the local Berry curvature, however the contributions from K and K’ points for individual bands are opposite and cancel each other. Accordingly for the Fermi energy located in the band gap, the total Berry curvature is zero, thus also the total Chern number for the fully occupied valence bands is zero. As a result, the anomalous Hall effect does not occur, as should be in systems with the time reversal symmetry, but one can observe for the Fermi level inside the energy gap, finite and quantized valley Hall conductivity. It is worth noting that the valley Hall effect can be detected experimentally, e.g. with the use of the Kerr rotation microscopy^62,63^.

Fig. 6 present the valley Hall conductivity as a function of Fermi energy and for specific twist angles for all four heterostructures considered in this paper. All figures have been ploted based on analytical formulas obtained based on Eq. (22) however, as they are rather long and awkward we decided not to show them. The valley Hall conductivity is a symmetric function with respect to change of the sign of Fermi energy and reveals well defined plateau when the Fermi level is located inside the energy gap. Importantly, the valley Hall conductivity takes either +2 or -2 conductance quanta depending on the choice of the twist angle.

**Fig. 7:**
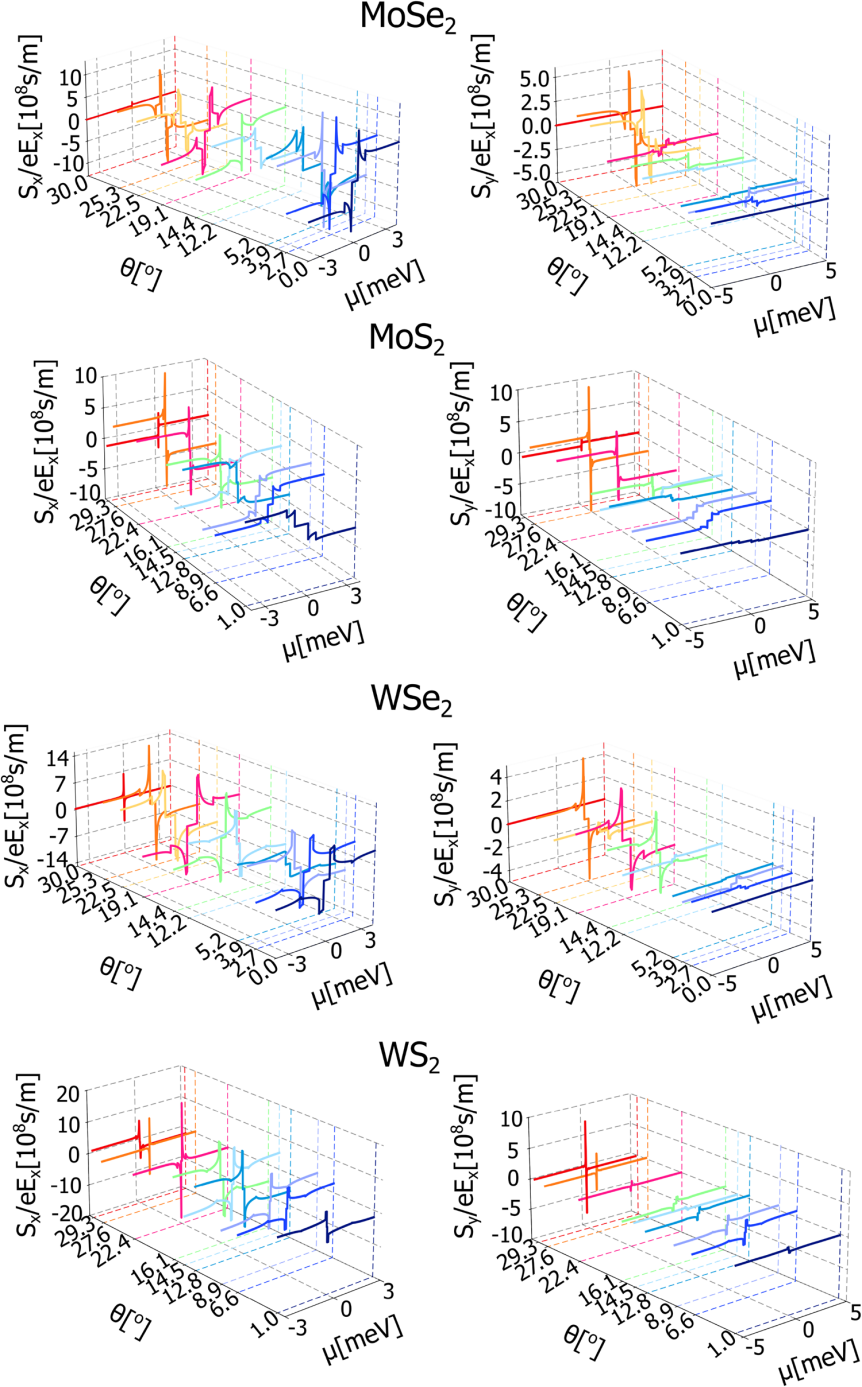
The *x* and *y* components of nonequilibrium valley spin polarization plotted as a function of Fermi energy, $\mu$, for specific values of the twist angle, $\theta$. The values of parameters $\lambda$, $\lambda _{R}$, $\phi _R$ and $\Delta$ for the certain twist angle, $\theta$, are taken from Fig 1, the $\upsilon$ parameter is equal $5.414\cdot 10^{-10}$ eVm for WSe$\phantom{0}_2$, $4.348\cdot 10^{-10}$ eVm for WS$\phantom{0}_2$, $5.413\cdot 10^{-10}$ eVm for MoSe$\phantom{0}_2$, and $4.356\cdot 10^{-10}$ eVm for MoS$\phantom{0}_2$.

Finally, we have considered the valley Rashba Edelstein effect, that is a valley nonequilibrium spin polarization that can be defined as^42,43,54,64^:

24 $$\begin{aligned} S_{\alpha }^{V} = S_{\alpha }^{K}-S_{\alpha }^{K'}, \end{aligned}$$

where $S_{\alpha }^{\nu }$ can be found based on Eq. (20). It has a meaning of emerging nonequilibrium spin imbalance between contribution of the graphene quasiparticles assigned to the two distinct valleys. Fig 7 presents the valley Rashba Edelstein effect plotted as a function of Fermi energy for certain twist angles. The valley spin polarization, similarly as the total spin polarization, is also aligned in plain of the heterostructure and is proportional to the external electric field. It strongly depends not only on the twist angle but also on the Rashba angle, as it is sensitive to the the spin-momentum locking in the structure. Importantly Valley spin conductivity does not depend on the relaxation time, thus it is roboust to the effects of impurities and other disorder responsible for the relaxation proces in the heterostructure.

## Discussion and summary

We have considered graphene deposited on four different semiconducting TMDC monolayers in the 2H phase, i.e., on MoSe$\phantom{0}_2$, WSe$\phantom{0}_2$, MoS$\phantom{0}_2$, and WS$\phantom{0}_2$. The main objective was to check how the relative twist angle between graphene and semiconducting TMDC monolayer changes spin-orbital proximity effects and orbital physics in the electronic states of graphene. Accordingly, using effective low-energy Hamiltonian and Green function formalism we calculated the spin Hall effect and nonequilibrium spin polarization in the system. In both cases we have derived analytical formulas, which clearly show that the twist angle can strongly modulate both spin Hall and spin-to-charge conversion characteristics. One can conclude that the spin Hall effect and nonequilibrium spin polarization in the considered heterostructures originate from the proximity-induced Rashba and valley-Zeeman spin-orbit couplings. In consequence, the z component of spin is not conserved and therefore spin Hall conductivity does not reveal the quantum spin Hall insulator phase, when the Fermi energy is located in the energy gap. As the staggered potential and intrinsic spin-orbit coupling are significantly modulated by the twist angle, $\theta$, both the spin Hall conductivity and Rashba-Edelstein effect are modulated by the twist, as well. Importantly, the spin Hall effect does not depend on the Rashba angle, $\phi$, whereas the Rashba-Edelstein effect is substantially affected by the angle $\phi$, what results in an additional component to the spin polarization which is parallel to the external electric field. We have also determined the valley Hall effect and valley Rashba-Edelstein effects, and have shown that both valley transport phenomena are strongly modulated by the relative twist between graphene and TMDC monolayers. This is natural consequence of the fact that the twist angle strongly modulates the orbital-dependent characteristics and also the local contributions to the Berry curvature. Importantly we have shown that the valley Hall conductivity can achieve the quantized value equal to $\pm 2 e^2/h$.

It should be noted that in our calculations we have assumed low concentration of short-range nonmagnetic impurities. In other words, the calculations have been done in the constant relaxation time approximation, i.e., the relaxation time, $\tau$, is treated as a constant parameter, and we have not included the impurity vertex correction. It is well known that the impurity vertex correction has a remarkable impact on the spin Hall conductivity in systems with Rashba interaction and can lead to suppression of the spin Hall effect^65–68^. The issue of vertex correction is more complex in graphene-based heterostructures, where the spin-orbital proximity effects induce, apart of the Rahba coupling, also the valley-Zeeman interaction, anisotropic spin-orbital terms and spatial fluctuations of spin-orbital fields. It has been shown, for example, that the presence of valley-Zeeman term, in coexistence with Rashba SOC, leads to a nonvanishing spin Hall effect upon the vertex correction. The vertex correction in graphene-based heterostructures has been discussed in detailed in the context of both nonequilibrium spin polarization and spin Hall effect for example in^50,67–69^. Detailed evaluation of the impact of disorder on relaxation time and transport characteristic in graphene-based twisted structures is an important issue and some results on this have been reported recently^70,71^. However the effect of disorder in the structures considered in this paper requires a comprehensive consideration that treats on the equal footing the impurities (including spin-orbit one) and fluctuating in space Rashba and intrinsic spin orbit interactions. Accordingly, to emphasize topological aspect, we decided to restrict considerations presented in this paper only to the clean limit. The more comprehensive theoretical description the effect of the twist angle on the relaxation processes and vertex correction will be considered in the future paper.

In the end it should be stressed that, the physics of spin-to-charge conversion effects upon twist could be much richer in case of graphene deposited on other materials. For example deposition of graphene on semiconducting TMDCs monolayers in the T structural phase leads to the crystal-field-induced in-plane anisotropic valley-Zeeman and Kane-Mele terms. Importantly, the anisotropic Kane-Mele term opens a topological energy gap and one can expect the quantum spin Hall effect, even in the presence of large Rashba SOC^72^. Graphene grown on top of a Pb superstructure also shows the in-plane valley-Zeeman type of SOC^73^. In turn, graphene deposited on group IV monochalcogenides reveals novel Rashba terms^74^. All these examples show that the physics of proximitized graphene is extremely rich and still needs detailed, comprehensive studies. Whereas the twisting of graphene with respect to substrates can introduce more complexity, but it may also be a useful tool for controlling and tuning the proximity effects and therefore spin-to-charge conversion and valley contrasting physics in graphene-based structures.

## Data Availability

All data that support the findings of this study are included within the article.
